# Sustainable Production of Dental and Orthodontic 3D Models Through Fused Granular Fabrication of Recycled Polymers

**DOI:** 10.3390/bioengineering13050558

**Published:** 2026-05-15

**Authors:** Jens Kruse, Malte Stonis, Julia Barasinski, Florian Konstantin Stangl, Hisham Sabbagh

**Affiliations:** 1 IPH— Institut für Integrierte Produktion Hannover gGmbH, 30419 Hanover, Germany; 2 Department of Orthodontics and Dentofacial Orthopedics, LMU University Hospital, 80336 Munich, Germany

**Keywords:** recycling, orthodontics, dentistry, fused granular fabrication, 3D printing, additive manufacturing

## Abstract

Sustainable production in dental and orthodontic 3D printing has gained increasing attention due to environmental concerns and the need for cost-effective and resource-saving solutions. This study presents a proof of concept for using recycled polymers and fused granular fabrication (FGF) in a closed-loop 3D printing approach, omitting intermediate filament manufacturing. A desktop 3D printer served as the kinematic platform and was modified with a pellet-based extruder to directly process recycled polyethylene terephthalate glycol (PETG) flakes, obtained by shredding previously printed PETG parts, into dental models. Dimensional accuracy was evaluated using optical 3D scanning analysis. The results indicate that models produced from recycled PETG are, in principle, suitable for dental and orthodontic applications within the investigated scope. This technical note provides initial evidence supporting the integration of recycled thermoplastics into dental and orthodontic model fabrication as part of sustainable additive manufacturing workflows. Potential pathways for workflow integration in clinical and laboratory environments, as well as directions for future research, are outlined, including the optimization of printing parameters and process stability. The main technical challenges were unreliable feedstock flow, causing bridging and jamming, while thermal creep from insufficient inlet cooling promoted premature softening of the flakes, causing torque spikes and unstable feeding.

## 1. Introduction

Additive manufacturing (AM), commonly referred to as 3D printing, has been widely adopted in the medical field for the fabrication of patient-specific products due to its precision and efficiency [1,2,3]. In orthodontics, AM has revolutionized fabrication processes across the entire laboratory spectrum, including diagnostic dental models, surgical guides, treatment appliances and models for aligner therapy [4,5]. Despite its benefits, AM relies extensively on single-use plastics and resins. These resins are typically non-recyclable due to their chemical composition and contamination issues, thus significantly contributing to environmental waste [6,7]. The widespread adoption of aligner therapy in orthodontics results in the disposal of tens of millions of non-recyclable plastic models annually, raising awareness with regard to the impact on the environment [8,9]. Reflecting global concerns, 127 nations have already enacted legislation to regulate and reduce avoidable single-use plastics [10,11,12]. In the context of sustainable production and legal considerations, the increasing demand for eco-friendly alternatives has driven research into the use of recycled materials [13,14,15]. Despite these efforts, recycling rates remain relatively low, with less than 11% of plastic waste recycled in Europe as of 2020 [16]. AM technologies, such as fused filament fabrication (FFF) and fused granular fabrication (FGF), enable the use of recyclable thermoplastics like polyethylene terephthalate glycol (PETG). These methods offer promising strategies for reducing waste and promoting sustainable manufacturing compared to non-recyclable techniques like Stereolithography (SLA) and Digital Light Processing (DLP) [8,17]. Instead of filament for the FFF process, FGF 3D printing uses granules or pellets as feedstock, reducing costs and enabling easier material distribution [18]. The application of FGF is particularly promising for producing dental diagnostic models and orthodontic aligner models, given the high frequency of their use, large-scale production, and minimal risk of contamination [19]. These models are typically utilized for documentation, diagnostic analysis, or produced as manufacturing byproducts. Nevertheless, achieving the necessary print quality, dimensional accuracy, mechanical characteristics, and biocompatibility must be ensured. This study aims to detail modifications made to a pellet extruder designed specifically for FGF-based 3D printing and to provide initial indications of its suitability for the sustainable production of additively manufactured dental models from recycled PETG. In contrast to conventional workflows, recycled PETG was processed directly after shredding, without pelletizing, using 1–3 mm flakes as granular feedstock. Prior studies show PETG to be well suited for mechanical recycling in AM, retaining good processability and largely preserved mechanical and thermal properties over multiple cycles. Reported degradation effects are moderate, supporting closed-loop use in both filament- and pellet-based extrusion workflows [20,21]. Thermomechanical recycling induces non-monotonic property changes: initial increases in stiffness and strength due to chain rearrangement and crystallization may occur before chain scission dominates at higher recycling counts. Spectroscopic analyses indicate progressive molecular weight reduction, while thermogravimetric data show largely unchanged degradation onset temperatures. Overall, these findings confirm the feasibility of recycled PETG for material-extrusion-based additive manufacturing, provided the number of recycling cycles is controlled.

## 2. Materials and Methods

### 2.1. Experimental Setup and Modified Pellet Extruder

**Figure 1:**
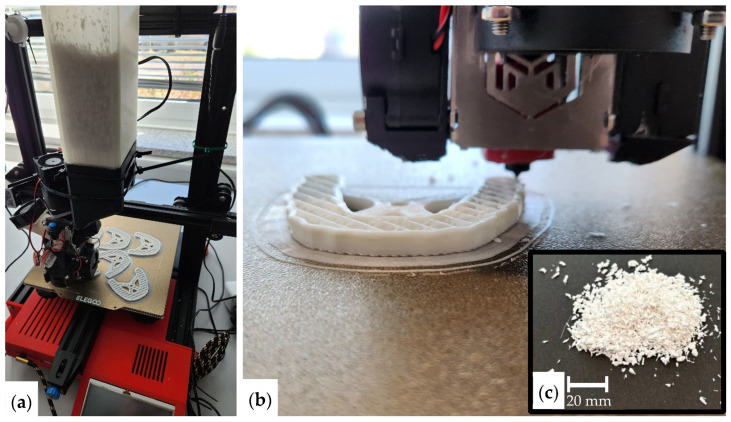
(**a**) Test stand for FGF 3D printing, (**b**) close-up of the FGF printing process, and (**c**) PETG flakes.

**Figure 2:**
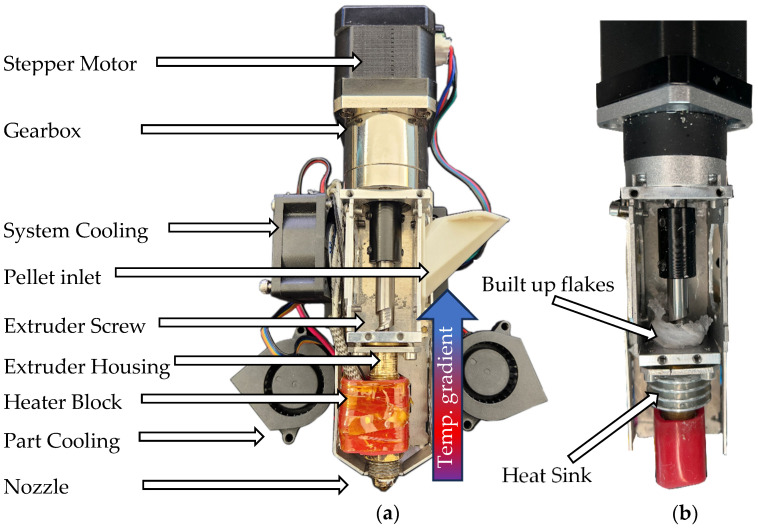
(**a**) Mahor XYZ V4 Pellet Extruder in stock configuration, (**b**) with additional heat sink and clogged-up material inlet.

The experimental setup is based on a desktop 3D printer (Creality Ender 3 V2, Shenzhen Creality 3D Technology Co., Ltd., Shenzhen, China), which served as the kinematic platform (Figure 1). Instead of the standard filament-based print head, the system was equipped with the Model V4 Pellet Extruder (Figure 2) by IAMTECH 2019 S.L.U. (trading as MAHOR.XYZ, Navarra, Spain).

The extruder is mounted on the X-axis via a quick-change tool system (Hermit Crab V1, Shenzhen BIQU Technology Co., Ltd., Shenzhen, China), enabling rapid exchange and adaptation of the print head for experimental purposes. The main parameters of the modified printer setup are summarized in Table 1.

Pellets suitable for FGF can be obtained from various manufacturers. In this study, PETG pellets supplied by DAS FILAMENT (Roman Stieben, Emskirchen, Germany) were initially used to fabricate printed parts. These parts were subsequently mechanically recycled using a QiTech JARIV industrial shredder, producing PETG flakes (Figure 1) that were directly utilized as granular feedstock for the FGF experiments presented in this work. The feedstock material is gravity-fed from a hopper into the extruder’s inlet (Figure 2a). A steel screw conveys the material through a brass housing to the nozzle. At the end of the extrusion screw, a heating block with a 70 W cartridge heater is installed. The material then gets extruded out of a copper-plated 0.4 mm nozzle.

This printer–extruder combination had previously been used at IPH to successfully process recycled PLA. Furthermore, according to the manufacturer’s documentation, ABS has also been processed successfully [22,23]. Given that PETG’s processing temperature range (approximately 230–250 °C) lies between that of Polylactic Acid (PLA) (~200 °C) and Acrylonitrile–Butadiene–Styrene (ABS) (~250 °C), its suitability for pellet-based extrusion was initially assumed [24,25,26]. However, during preliminary tests with recycled PETG, recurrent build-up of partially molten material was observed at the screw inlet, even after the installation of an additional heat sink (Figure 2b). For the experiments and workflow presented in this paper, the inlet region was manually inspected and cleaned regularly between prints to ensure uninterrupted operation and to minimize interference caused by thermal creep. The heat sink on the upper part of the barrel therefore remained in place throughout the experiments; however, no active cooling was applied. The troubleshooting of thermal creep and potential future solutions to mitigate this phenomenon are discussed later in this work.

### 2.2. Slicing and 3D Printing

A mandibular dental model was designed using orthodontic imaging software (OnyxCeph3TM, Image Instruments, Chemnitz, Germany) and exported in STL format. OrcaSlicer (V2.3.0-rc) was used to prepare the geometries for the AM process and to define process-specific parameters. The software, derived from the open-source project Slic3r, integrates functionalities from PrusaSlicer (Prusa Research a.s., Prague, Czech Republic) and Bambu Studio (Bambu Lab, Shenzhen, China) [27]. The slicing parameters used are summarized in Table 2. The printing was conducted at room temperature within the 3D Printing Lab at IPH. Humidity and temperature within the room were not controlled. The printed parts were measured after being cooled down to ambient air temperature.

### 2.3. Optical Measurements

For three-dimensional acquisition of the printed geometries, an optical measurement system (ATOS Core 500, GOM GmbH, ZEISS Group, Braunschweig, Germany) was used. The system uses structured light projection and stereo camera triangulation, enabling contactless surface digitization of medium-sized components. The selected sensor configuration provides a measuring volume of 500 × 380 mm at a working distance of 440 mm, with a point spacing of 65 µm accuracy. Dimensional evaluation and comparison between the digital reference file and the printed model were conducted using GOM Inspect 2016. The parts were scanned from multiple orientations to ensure adequate surface coverage and data quality, in accordance with previously reported procedures demonstrating reliable and repeatable acquisition performance [28,29].

The printed model was scanned once prior to and once after thermoforming of an orthodontic aligner (CA Pro+, Scheu-Dental GmbH, Iserlohn, Germany) to assess whether the thermoforming process induced irreversible deformation or warping of the thermoplastic model and to evaluate its dimensional stability for potential use in aligner manufacturing. The aligner was fabricated according to the manufacturer’s instructions using a pressure molding machine (Biostar, REF 3001; Scheu Dental GmbH, Iserlohn, Germany). The sheets were heated to 220 °C and then pressure-molded onto the models under a pressure of 4.9 bar for 30 s. After cooling to room temperature, aligners were removed, and the models were rescanned to assess potential irreversible geometric changes induced by the thermoforming process.

The GOM Inspect 2016 software facilitated mesh inspection, alignment, deviation analysis, and documentation of deviations between target and manufactured geometries.

## 3. Results

**Figure 3:**
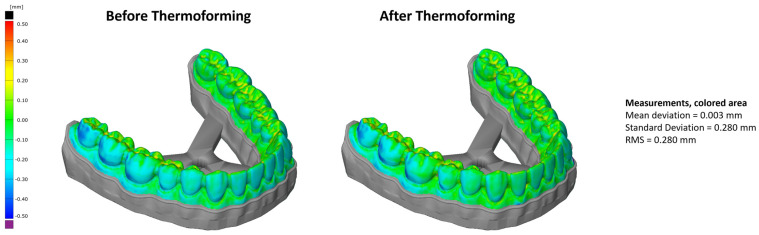
Optical deviation analysis between the digital reference (CAD) and the printed model (FGF): (**left**) before thermoforming; (**right**) after thermoforming.

The results from the comparison of the reference geometry with the scanned printed models, both before and after thermoforming, are shown in Figure 3. Only the clinically relevant region for aligner thermoforming was considered in the deviation analysis; support structures and basal regions were excluded from evaluation. This was done via the tools within the GOM Inspect Software (versions 8 and 2016). Deviations remained within ±0.25 mm in most clinically relevant areas for both conditions, except for the transversal support bar and for localized regions where reference markers had been temporarily attached to the model (mean deviation of 0.003 mm, standard deviation of 0.280 mm, and RMS of 0.280 mm). Characteristic tooth segments exhibited distinct geometric behavior: Molars, characterized by broad fissured surfaces and bulky morphology, remained largely dimensionally stable. Canines, which are tall and narrow with pointed cusps, showed minor localized deviations. In contrast, incisors, being thin and mechanically slender, exhibited the highest susceptibility to deformation, particularly at the incisal edge.

## 4. Discussion

This study explored the feasibility of using recycled PETG for the sustainable production of 3D models through a fused granular fabrication approach without further processing the shredded material. The results demonstrate that flakes from recycled PETG can be processed effectively to produce dental and orthodontic models with acceptable dimensional accuracy. The modified extrusion system, based on a desktop 3D printer modified for granulate processing, confirmed the technical viability of FGF and highlighted its potential as a sustainable alternative to conventional 3D printing technologies such as SLA and DLP. Compared to FFF, FGF enables direct use of shredded material, eliminating filament extrusion and thereby reducing thermomechanical degradation, energy consumption, and processing costs [18]. By minimizing additional melting and handling steps, FGF supports a more resource-efficient workflow while maintaining material performance, highlighting its suitability for closed-loop recycling of PETG in dental and orthodontic applications. Given the widespread use of AM for the production of dental and orthodontic models and the resulting generation of tens of millions of single-use resin models annually, the adoption of recyclable alternatives such as PETG presents significant ecological and economic advantages [30,31]. The following sections discuss the dimensional accuracy results, technical challenges encountered, the implementation pathways for clinical and centralized workflows, and the outlook for future development.

### 4.1. Dimensional Accuracy

Dimensional analyses through optical 3D scanning confirmed that the printed models consistently met the clinical accuracy threshold of ±0.25 mm [32,33]. The observed differences in dimensional deviations between molars, canines, and incisors can be plausibly explained by the interplay of geometric characteristics and thermomechanical shrinkage behavior inherent to material-extrusion-based additive manufacturing. Molars feature compact, volumetric geometries with relatively low surface-to-volume ratios, which promote slower and more homogeneous cooling, thereby reducing localized thermal gradients and associated shrinkage effects. In contrast, incisors exhibit slender geometries with thin cross-sections and pronounced edges, resulting in faster cooling rates, higher thermal gradients, and increased sensitivity to anisotropic shrinkage and warping. Canines represent an intermediate case, combining elongated morphology with localized mass at the cusp, which may explain the limited and spatially confined deviations observed. Within the FGF process, these effects may be further amplified by layer-wise heat accumulation, directional deposition paths and material-specific thermal properties of PETG, such as its relatively low glass transition temperature combined with a high processing temperature.

Consequently, tooth-specific deviation patterns appear to be governed not by positional factors alone, but by the interaction of geometry-dependent cooling behavior and thermally induced shrinkage mechanisms. While this mechanistic interpretation remains qualitative, it offers a plausible explanation for the localized accuracy variations observed in recycled-PETG dental models. These findings also underline the need for further studies that combine geometric feature analysis, thermal monitoring and process simulation to better understand and control these deviations.

### 4.2. Technical Challenges and Process Limitations

**Figure 4:**
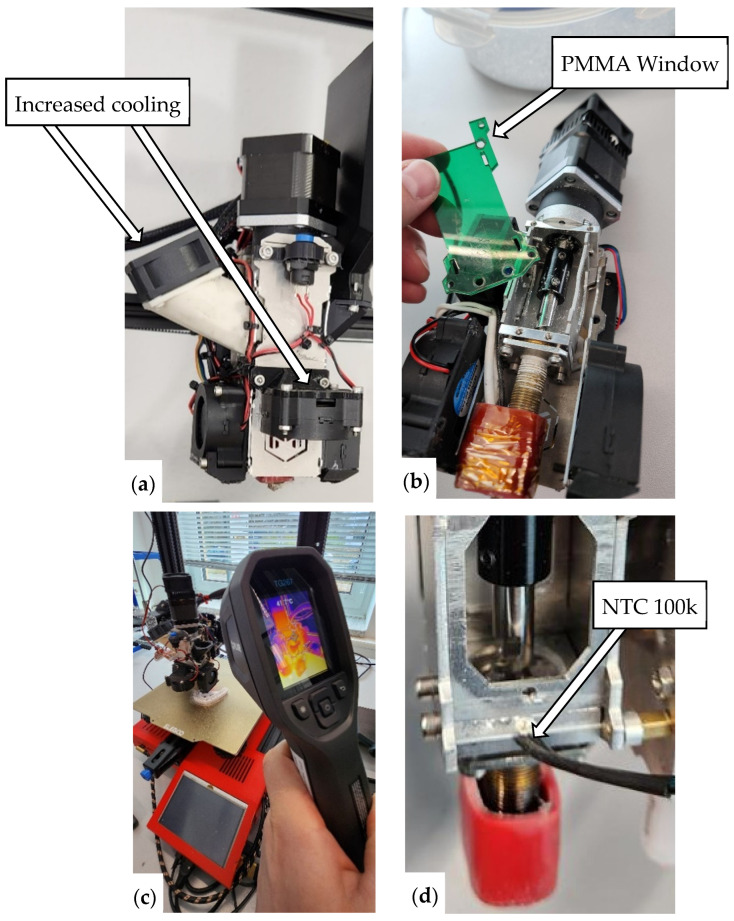
(**a**) Actively cooled heat sink and side-mounted 40 mm fan for inlet zone, (**b**) PMMA panel to examine process, (**c**) thermal analysis of heat creep, and (**d**) NTC 100 k to measure inlet-zone temperature.

Despite the viability of the method, several technical challenges were identified, particularly related to thermal management within the pellet extruder. The large temperature difference between PETG’s glass transition temperature (approximately 75 °C) and its printing temperature (around 250 °C) led to significant heat transfer from the heating block into upstream sections of the extruder. This caused premature softening of the feedstock, clogging, and irregular flow behavior. This effect was not problematic with PLA (190–210 °C) but consistently occurred with PETG after long print durations or when starting a new print before the extruder had fully reheated. If left unaddressed, the material build-up eventually blocked the inlet, halting extrusion. In several cases, this led to mechanical failure of the extruder’s planetary gearbox, likely due to jamming and overloading from increased torque at low extrusion temperatures. Noticeable gear wear confirmed this diagnosis. As part of the feasibility evaluation, various cooling strategies were tested to mitigate this issue. Figure 4a shows the modified extruder with a new stepper motor (NEMA17 1:5.18 geared) and enhanced cooling for the inlet region. A transparent PMMA panel (Figure 4b) was temporarily added to monitor pre-melting behavior visually. Despite these modifications, material blockage still occurred intermittently.

It became evident that extrusion behavior was strongly governed by the temperature difference (ΔT) between the material’s printing temperature and its glass transition temperature (T_g_). For PLA (T_g_ ≈ 60 °C, T_print_ ≈ 190–200 °C) and ABS (T_g_ ≈ 100 °C, T_print_ ≈ 250 °C), ΔT typically ranges from 130 to 150 K, whereas the PETG used in this study (T_g_ ≈ 75 °C, T_print_ ≈ 250 °C) exhibits a substantially larger ΔT of approximately 175 K.

Consequently, the higher processing temperature required for PETG, combined with its lower T_g_ compared to ABS, demands a steeper temperature gradient between the nozzle and the screw inlet, a condition that could not be sufficiently maintained, even with additional cooling measures. As a result, operation at a nozzle temperature of 250 °C (compared to ~200 °C for PLA) likely intensified heat conduction from the heater block into upstream regions of the screw (compare Figure 2a). This led to premature softening of the PETG flakes as local temperatures exceeded their T_g_, causing recurrent material accumulation at the screw inlet and subsequent clogging of the extrusion path (Figure 4).

To qualitatively evaluate this assumption, a 100 kΩ NTC thermistor was installed on the aluminum housing near the screw inlet (Figure 4d). During PETG extrusion, surface temperatures of up to 65 °C were measured at this location—close to T_g_ of PETG. Although these values do not represent the internal temperature of the screw, they indicate substantially elevated thermal conditions near the inlet, suggesting even higher temperatures closer to the screw due to limited heat dissipation of the housing. These measurements support the hypothesis that thermal creep was the primary cause of the observed extrusion instability.

While manual cleaning between prints temporarily mitigated clogging, such interventions limit the practical applicability of the system in automated or industrial production environments. Although the slicing process using OrcaSlicer enabled adequate customization of machine and material parameters, it provided limited means to compensate for extrusion instability due to heat creep. Overall, the requirement for frequent manual adjustments during printing highlights the limited robustness of current FGF implementations for PETG when using this extruder design. In subsequent trials, a stirrer mechanism was installed in the inlet region to prevent agglomeration and to ensure continuous motion of the PETG flakes. In addition, external vibration motors were tested, and periodic motion patterns were introduced via G-code—such as oscillatory print-head movements every few layers—to promote consistent feedstock flow and to avoid prolonged residence of the material in thermally critical regions. However, none of these measures proved effective in preventing premature softening and material build-up at the screw inlet during extended operation. Therefore, in future work, different extruder designs will be investigated for the usage of PETG flakes. By comparison, PLA flakes could be printed reliably over several hours without intervention. PETG flake-based prints, however, were only successful when the feedstock was manually stirred and intermittently checked, indicating a substantially lower process robustness for PETG under otherwise similar conditions. As this work was intended as a proof-of-concept study and did not include extensive experimental series, no statistical evaluation of these observations can be provided at this stage.

Based on the authors’ previous work [34] on thermomechanical recycling in material-extrusion-based AM, it is well established that material behavior and degradation over multiple recycling cycles must be carefully considered for long-term and scalable applications. In particular, prior studies have shown that polymers such as polypropylene (PP) and thermoplastic elastomers (TPEs) can be recycled and processed additively over several cycles, while exhibiting only moderate changes in thermal and rheological properties, albeit with gradual effects on mechanical performance and printability. These findings demonstrate the fundamental suitability of PP and TPE for repeated recycling in both filament-based and pellet-based AM workflows, while simultaneously highlighting that progressive degradation and process stability become increasingly relevant factors with rising recycling counts.

Against this background, the present study deliberately focused on a single recycling loop of PETG as a proof of concept, prioritizing the assessment of feasibility, dimensional accuracy, and process-related limitations in the used FGF process. While no conclusions on long-term degradation behavior can be drawn from the current data, insights from previous work underscore the necessity of future investigations addressing recycling-induced material changes, including viscosity evolution, thermal stability, and extrusion robustness, to enable reliable closed-loop workflows in dental and orthodontic applications.

### 4.3. Implication for Clinical Application and Sustainability

The findings of this study support the conceptual development of a closed-loop recycling workflow using PETG for the sustainable production of dental and orthodontic models. In such a system, printed models are used and subsequently shredded into flakes, which are then reused for AM. This approach offers a promising pathway to reduce plastic waste, particularly in light of the millions of non-recyclable models currently discarded each year as part of aligner therapy and other digital workflows [8,9]. For practical implementation and scalability, the shredding and reprocessing stages are best centralized, where material handling, quality control, and pre-treatment can be performed under controlled conditions. However, even under optimized conditions, repeated recycling cycles may result in polymer degradation and contaminant accumulation, which can negatively impact mechanical performance, surface finish, and extrusion stability [35,36]. Defining the acceptable number of recycling loops and establishing effective pre-treatment protocols will be critical to ensuring material consistency and long-term process reliability in FGF. Nonetheless, FGF can be applied in-office using recycled PETG. Modifying standard desktop 3D printers to accommodate pellet-based extrusion is cost-effective and accessible, potentially allowing smaller clinics or laboratories to participate in local closed-loop workflows. Maintaining consistent print quality in such settings would depend on access to reliable pellet material and routine equipment maintenance.

For clinical application, specifically for aligner manufacturing, future studies should evaluate not only the dimensional accuracy and thermal stability of the printed models, but also the geometry, fit, and seating behavior of the resulting aligners.

## 5. Outlook

For broader adoption, several areas require further investigation and development. From an environmental and economic standpoint, closed-loop AM could significantly reduce plastic waste in dentistry and orthodontics. In terms of technology development, further optimization of extrusion hardware and process standardization is necessary to improve thermal stability, material handling, and long-term printing reliability. Additionally, future work should include systematic documentation of material moisture content, ambient air humidity, and room temperature to better assess their influence on process stability. Investigating the lifecycle performance of recycled PETG, including the degradation curve over multiple cycles, is part of an ongoing research project. Additionally, future work may more specifically explore the suitability of the FGF method for aligner manufacturing in comparison with the method of directly printing aligners as an alternative to the model-based thermoforming process. Regarding this comparison, toxicological considerations are of central interest due to the possible leaching of residual monomers (GC/MS and cell culture).

## 6. Conclusions

Recycled PETG flakes were successfully processed via fused granular fabrication to produce dental and orthodontic models with clinically acceptable accuracy and surface quality. The results confirm the feasibility of this approach as a sustainable alternative to conventional resin-based workflows. Implementing closed-loop recycling systems for PETG could substantially reduce plastic waste and material costs in dental and orthodontic model fabrication.

## Figures and Tables

**Table 1:** Parameters of the modified Creality Ender 3V2 3D printer with Mahor XYZ Pellet Extruder V4.

Parameter	Specification
Mainboard	Octopus V1.1, Shenzhen Big Tree Technology Co., Ltd. (BIGTREETECH), China
Printer Base	Creality Ender 3 V2, Shenzhen Creality 3D Technology Co., Ltd., China
Firmware	Klipper (up to version 0.12.×)
Nozzle	0.40 mm, E3D V6, copper, nickel-plated
Extruder	Pellet Extruder V4, MAHOR.XYZ (IAMTECH 2019 S.L.U.)
Extruder Motor	NEMA17 geared stepper motor, 1:5.18 reduction ratio
Feedstock	Recycled PETG granules/flakes from shredded prints (FGF)
Max. Extrusion Temp.	300 °C
Heating Power	70 W @ 24 V
Available Build Volume	Approx. 150 × 150 × 150 mm (reduced due to pellet extruder)

**Table 2:** Slicing parameters for FGF 3D printing of dental models.

Parameter	Value/Setting
Material	Recycled PETG flakes (FGF feedstock)
Nozzle Temperature	250 °C
Bed Temperature	75 °C
Layer Height	0.12 mm (first layer: 0.20 mm)
Nozzle Diameter	0.40 mm
Extrusion Widths	Perimeter: 0.45 mm; Infill: 0.60 mm; Top: 0.40 mm
Filament Diameter (virtual)	1.75 mm (only for flow calculation, FGF mode)
Retraction Settings	1.5 mm @ 60 mm/s
Z-Hop Height	0.2 mm
Cooling	Part cooling fan: 100%; Additional: 70%
Infill Pattern/Density	Crosshatch/15%
Printing Speed (outer walls)	40 mm/s
First Layer Speed	15 mm/s
Estimated Print Time	2 h 38 min
Material Usage	ca. 21.91 g (17.67 cm^3^)
Build Volume (Z height)	Up to 19.52 mm
Print Profile	Custom (FGF-Ender with 0.4 mm nozzle)

## Data Availability

The original contributions presented in the study are included in the article, further inquiries can be directed to the corresponding author.
